# Robust Sparse Representation and Multiclass Support Matrix Machines for the Classification of Motor Imagery EEG Signals

**DOI:** 10.1109/JTEHM.2019.2942017

**Published:** 2019-10-02

**Authors:** Imran Razzak, Ibrahim A. Hameed, Guandong Xu

**Affiliations:** 1 University of Technology 226156 SydneyNSW2007Australia; 2 Norwegian University of Science and Technology 8018 7491TrondheimNorway

**Keywords:** Brain-computer interfaces, Electroencephalography (EEG), Principal component Analysis (PCA), Brain disorder

## Abstract

Background: EEG signals are extremely complex in comparison to other biomedical signals, thus require an efficient feature selection as well as classification approach. Traditional feature extraction and classification methods require to reshape the data into vectors that results in losing the structural information exist in the original featured matrix. Aim: The aim of this work is to design an efficient approach for robust feature extraction and classification for the classification of EEG signals. Method: In order to extract robust feature matrix and reduce the dimensionality of from original epileptic EEG data, in this paper, we have applied robust joint sparse PCA (RJSPCA), Outliers Robust PCA (ORPCA) and compare their performance with different matrix base feature extraction methods, followed by classification through support matrix machine. The combination of joint sparse PCA with robust support matrix machine showed good generalization performance for classification of EEG data due to their convex optimization. Results: A comprehensive experimental study on the publicly available EEG datasets is carried out to validate the robustness of the proposed approach against outliers. Conclusion: The experiment results, supported by the theoretical analysis and statistical test, show the effectiveness of the proposed framework for solving classification of EEG signals.

## Graphical abstract

The aim of this work was to design an efficient approach for robust feature extraction and classification for the classification of EEG signals. We applied robust joint sparse PCA (RJSPCA) and Outliers Robust PCA (ORPCA) and compared their performance with different matrix base feature extraction methods, followed by classification through support matrix machine. The combination of joint sparse PCA with robust support matrix machine showed good generalization performance for classification of EEG data due to their convex optimization.

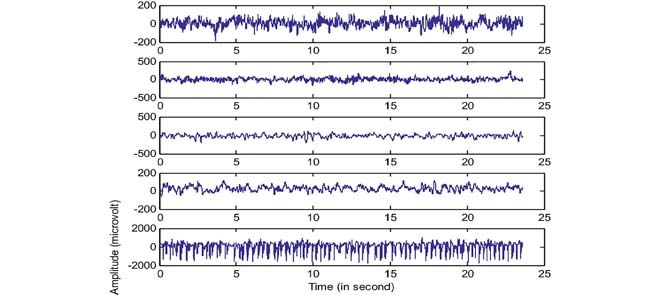

## I. Introduction

Electroencephalography (EEG) signals are electrophysiological monitoring recording of electrical potentials to capture the activity of the brain. Clinically, it refers to the signals of brain’s spontaneous electrical activity over a short period of time. EEG signals analysis has been actively used by clinicians to identify abnormalities in human brain such as depth of anesthesia, coma, sleep disorders, encephalopathies and brain death etc. Early days, the investigations were based on visual inspection by trained clinical, thus are prone to error, qualitative and require extensive training. The advancement in data acquisition devices and computer technology have made it possible to identify abnormalities successfully [27], [29].

EEG signals are extremely complex in comparison to other biomedical signals, thus requires efficient feature selection as well as classification approaches. Selection of important and discriminant features is the process of selecting useful subset of discriminant patterns. It is a key component for any machine learning problem, aiming to identify, a new unseen set of observation belong to which class based on the set of training samples that consist of known observations. Selection of discriminant patterns not only help to improve the classification accuracy and reduces the computational complexity but also helps to improve the generalization capability as well as alleviates the effect of the curse of dimensionality by reducing the dimensionality of the data [31], [33]. Traditional dimensionality reduction approaches such as Principal component Analysis (PCA), Linear discriminate Analysis (LDA) etc. could be used for dimensionality reduction, however, either these methods fail to select important pattern or show poor performance in the presence of outliers.

To overcome the complexity challenge involved due to high dimensionality of data, recently several methods based on vector data have been applied and different variants of these methods have been proposed to improve the performance against outliers such as PCA [32], [36], LDA [3], [28], LPP [7], SPP [25] and NPE [7] etc. Among these dimensionality reduction methods, PCA is one of the commonly used dimensionality reduction methods associated with multivariate analysis since its introduction by Pearson [22] and Hotelling [9]. It projects the input high dimensional data to linear orthogonal space that is much low in dimensionality as compare to original data, with the aim to sequentially extracts those uncorrelated orthogonal features that maximizes the variability of the data, thus it guarantee minimal loss of information. However, its major drawback is that PCA is a linear combination of all variables and loading (that are typically non-zero). There is additional cost involved in extraction and processing of each features [14]. Furthermore, there may be several features that are not important for potential application, that in results affect not only accuracy but computational performance. Thus, dimensionality reduction and selection of important pattern is very important, however, traditional methods are still sensitive to outliers [32]. Furthermore, most of the methods require to to reshape the data into vector form that present in the form of matrix, which results in losing the structural information exist in the original featured matrix that is very important for the task where such information is an important factor i.e. EEG signals consist of voltage fluctuations at several electrodes with respect to time, thus have strong correlation with respect to certain frequency band and channels. Thus, methods based on vector data fail to deal with such data i.e. data is required to be transform in to vector form before applying traditional methods for dimensionality reduction (i.e. PCA and LDA), consequently, such methods may not be able to exploit the embeded structural information very well in results affecting the performance. Recently, several variants of PCA have been presented to design robust dimensionality approach that not only able to select useful features but also robust against outliers [21].

Vector based methods have been successively applied for the high dimensional data classification and has shown promising classification performance. State-of-the-art vector based methods are (LDA) [24], [34], [40], support vector machines (SVM) [6], [10], [41], K nearest neighbor (KNN) [5], [16], [20]. However, with all these methods, we need to transform the matrix/tensor data in the form of vectors, thus affecting the classification performance due to the loss of structural information embedded in the data. An alternative approach to overcome this problem is to concatenate the matrix in to vectors before data classification which in turns in increasing the the data dimension that results in model over-fitting. To deal such data, recently researcher suppress the matrix into vectors using common spatial patterns [1], [13], [15], [17], [35], [39]. Although, these methods are able to deal with matrix/tensor data directly, however ignore the topological structure exist in the matrix, whereas considering such structural information provides an additional advantage to improve the classification performance.

Recently, sparse principal component analysis is being widely used to deal with the dimensionality challenge as well as to reduce the number of explicitly used variables. Inspired by perform of sparse PCA and its variants, in this paper, we performed feature selection using sparse methods and used support matrix machines as classifier. Results showed that sparse based dimensionality methods outperform the traditional dimensionality methods. Moreover, analysis shows that methods based on matrix directly, showed much better performance as compare to vector based methods. Compared to the state-of-art featured selection methods, the key contributions of this work as follows: Redundancy in the high dimensional data makes it a good candidate sparse representation, thus we utilized sparse principal component analysis and its variants for dimensionality reduction. Reshaping the data into vectors could ultimately destroy the structural information, thus, we have applied dimensionality reduction and classification methods directly on matrix data to preserve the structural information embedded in data. To validate the gain in performance, we have applied matrix based classification on be three benchmark EEG datasets. Experimental evaluation (discriminant features and classification) shows the considerable improvement in most cases.

## II. Materials and Methods

This study present a framework for the classification of epileptic EEG signals. The aim of this work is to explore the feature selection and classification methods based on matrix data. In this work, we investigates the robust feature extraction based on sparse component analysis (RJSPCA, ORPCA) and compared their perofrmance with state of the art feature selection methods. The feature extraction based on RJSPCA and ORPCA are robust against outliers and much efficient for dimensionality reduction. For classification purpose, we have used support matrix machines. Furthermore, we have used multiclass support matrix machines via maximizing the inter-class margin, that is computational efficient.

### A. Preprocessing

Motor imagery-based brain computer interaction translates the mental imagination of human movement into commands, consist of huge inter-subject variability with respect to the characteristics of signals of brain [2]. Moreover, EEG signals may consist of non-stationary and transitory behaviour such as measurement artifacts, outliers as well as non-standard noise in the EEG signal makes it difficult to classify. Due to the complexity of the EEG signals, we can not extract features directly. In order to overcome the variations involved in EEG signals, spatial filtering could be used as an effective approach to reduce the variability of EEG signals i.e. elimination of uncorrelated formations. We first need to determine the appropriate sample size of EEG signal. The desired sample unit is calculated as $n=\frac {Z^{2}p(1-p)}{e^{2}}$, where n is the number of sample size, p is an estimated proportion attributes, Z is the standard normal variate and e is the margin of error. In case of finite population, sample size can be calculated as $n=\frac {n}{1+(n-1)/N}$, where N is the size of population. In case if p is unknown, we have used 0.50 to produce the largest sample size. In this work, we have set the value of p = 0.50 for maximum sample size, and Z = 2.58, N = 4097 and e = 0.01 for 99% confidence level.

EEG data is stochastic and non-stationary, thus EEG signals are required to be stationary before classification. To make it stationary, we have divided the signals into several segments based on specific time interval in order to make it stationary for that short interval. We have segmented the EEG signals in to S number of sub sample based on T interval of time by defining S number of windows over EEG signals. In this experiment, we have divided the EEG signals into 4 windows (size of 1024, 1024, 1024 and 1025) with respect to time. In order to remove the artifacts as well as unrelated sensorimotor rhythms, in this work, we applied Filter Bank Common Spatial Pattern (FBCSP) algorithm [2] and perform autonomous selection of discriminative subject-specific frequency range in order to perfrm band-pass filtering of the EEG measurements. In order to select the dominant channels in each motor imagery, we applied CSP [15]. We further applied time domain parameters for feature selection [23] due to its proven robust performance [37], [42], [43]. The next task is allocation of sample size among S number of windows. We have calculated the best sample size for nth window OA.

\begin{equation*} n(i)=\frac {N_{i}\sqrt {\sum _{j=1}^{p} \mathrm {Var}_{i,j}^{2}}} {\sum _{i=1}^{k} N_{i}\sqrt {\sum _{j=1}^{p} \mathrm {Var}_{i,j}^{2}}}\tag{1}\end{equation*}

where n is the sample size required for ith window, Var is the variance of jth chanel in ith window.

### B. Feature Extraction

Feature selection is the process of choosing those pattern that are small in numbers as compare to original data and can best describe the data. It is the key component for any machine learning problem, aiming to identify, to which set of categories, a new unseen observation belongs on the basis of a training set of data containing known observations. It does not only help to increase the classification performance but also overcome the computational complexity. Moreover, it improves the generalization capability and alleviates the effect of the curse of dimensionality. Traditional feature extraction and classification methods require data to be transformed in the form of vectors before processing, which results in losing of structural information embedded in the original data matrix. In this work, we applied different variants of PCA on time domain parameters for efficient feature selection and dimensionality reduction.

Although principal component analysis and its different variants are able to improve the performance by overcoming the outliers to some extent, however the major disadvantage of these variants is that they even select those features that are already selected in other principal components thus feature are redundant. Furthermore, these methods are unable to select the required important features that could helps to improve the classification performance, however, selection of robust, discriminative and useful features could helps to improve the classification performance thus are important especially in the case, when selected features have some physical meaning. In order to overcome the redundancy, an alternative and simple approach is to consider the only loadings that are greater than threshold, however, it is inefficient and is not able to select important features. Sparsity could be achieved by imposing the $\ell _{0}$ coefficient on the regression coefficient which penalizes the number of non-zero coefficient whereas the loss term helps to minimizes the reconstruction error simultaneously.

\begin{align*}&arg\min _{A,B}= arg \min _{A,B} \| X-A^{T}BX\|_{F}^{2} + \lambda _{1} \|\beta _{j}\|_{0} \\&\text {subject to} \quad A^{T}A=I_{k}\tag{2}\end{align*}

The above objective function is able to determine informative features individually, however, it does not consider the structural relationship among mulitple features. SCoTLASS successfully derives sparse loadings using the lasso constraint in PCA, however, it is computationally inefficient, and lacks a good rule to pick tuning parameter [11]. To derive principal components with sparse loadings, several methods have been proposed to achieve the sparseness goal. Sparse PCA produces modified principal components with sparse loadings that are obtained by imposing the lasso constraint on the regression coefficients [45].

\begin{align*}&\hspace {-2pc}arg\min _{A,B}= arg \min _{A,B} \sum _{i=1}^{n} \| x_{i}-AB^{T}x_{i}\|^{2} \\&\qquad \qquad \qquad \quad + \lambda _{1} \sum _{j=1}^{k} \|\beta _{j}\|^{2} + \sum _{j=1}^{k}\lambda _{2,j}||\beta _{j}||_{1}\tag{3}\end{align*}

However, SPCA does not jointly select the useful features as $\ell _{1}$-norm is imposed on each transformation vector whereas $\ell _{1}$-norm is not able to select consistent features. In addition, $\ell _{2}$-norm is imposed on loss term, thus, it still suffers from outliers. Yi et al. presented JSPCA that select useful features jointly which helps to enhance the robustness of objective function against outliers [38]. In other words, JSPCA imposes the joint sparse constraints i.e., $\ell _{2,1}$-norm is imposed on loss term as well as on the regularization term respectively, to improve the robustness of algorithms.

\begin{equation*} arg\min _{B,A} J(B,A) = arg \min _{A,A} \| X-AB^{T}X\|_{2,1} + \lambda \|B\|_{2,1}\tag{4}\end{equation*}

Khan et al. presented joint group sparse PCA (JGSPCA) that ensure the group sparsity and forces the basic coefficient corresponding to a group of features to be jointly sparse [14]. The group sparsity ensure that the structurally integrity of the features. JGSPCA is able to select important features jointly and ensure the group sparsity, however, it is sensitive to outliers due to sensitivity of F-norm against outliers.

\begin{equation*} arg\min _{A,B} \!=\! arg \min _{A,B} \| X\!-\!\sum _{i=1}^{g} X_{\mathbb {G}}A^{T}B^{\mathbb {G}}\|_{F}^{2} \!+\! \lambda \!\sum _{i=1}^{g} \eta _{i}\|B^{\mathbb {G}}\|_{F}\tag{5}\end{equation*}

Razzak el al. introduced outliers robust two dimensional principal component analysis (ORPCA) by imposing the joint constraints. ORPCA relaxes the orthogonal constraints and penalizes the regression coefficient as a result ORPCA is able to selects important features and in the meantime it ignores the same features that have already be selected in other principal components [31], [32].

\begin{equation*} \min _{Q,P} J(Q,P) = \min _{Q,P} \sum _{j=1}^{N} \left \|{ X_{j} -X_{j} Q P^{T} }\right \|_{F}^{2} + \lambda _{a} \|Q\|_{F}^{2}\tag{6}\end{equation*}

Most of the existing methods, the projection involves the selection of all the original features thus there may be irrelevant and redundant features. Furthermore, considering the outliers presence in the data, to integrate selection of the features process into subspace to exclude redundant features and select optimal feature set, robust 2D-joint sparse PCA (2D-JSPCA) has freedom to jointly select the useful features as well as discard the features that already exist in other principal components [30]. It effectively combines the sparsity-inducing regularization and robustness of 2D-PCA by imposing the jointly sparse constraints on its objective function. The addition of penalty term makes the objective function robust against outliers as it penalizes all regression coefficient correspond to single feature as a whole.

\begin{align*}&\hspace {-1.5pc}\min _{Q,P} J(Q,P) = \min _{Q,P} \sum _{j=1}^{N} \left \|{ X_{j} -X_{j} Q P^{T} }\right \|_{F}^{2} \\&\qquad ~\qquad \qquad \qquad \qquad \quad + \lambda _{a} \|Q\|_{F}^{2} + \lambda _{b} \|Q\|_{2,1}\tag{7}\end{align*}

Besides intra-sample outliers, traditional classifiers either vector based or matrix based support machines are extremely fragile to the presence of outliers and are efficient to classify the corrupted data. Recently, low rank matrix completion methods have proven its important for exact matrix recovery from only partial of observation i.e. suppose we are given partially observed matrix, and we know that the full matrix can be decomposed as $X=L+S$, where matrix $L$ is low rank and S is sparse and consist of only few non zero columns. Here, both matrices $L$ and $S$ have arbitrary magnitude, rank of matrix $L$ as well as position and number of corrupted columns of matrix $S$ are unknown.

\begin{equation*} \min _{L,S} ||L||_{*}+\lambda ||S||_{1} \quad \text {s.t. } X=L+S,\tag{8}\end{equation*}

where $\lambda $ is the tuning parameter. RPCA helps to decrease the interclass variability. In RPCA, the low rank matrix L consist of meaningful features where as the sparse matrix S is the residual matrix tht consist of only few non zeros columns.

## III. Classification

The classical soft margin SVM is defined as

\begin{equation*} arg\min \frac {1}{2} tr(w^{T}w)+C\sum {1-y_{i}[tr(2^{T}x_{i})+b]}_{+}\tag{9}\end{equation*}

where ${1-y_{i}[tr(W^{T}X_{i})+b]}_{+}$ is the hinge loss, $W\in \mathbb {R}^{pq}$ is the vector of regression coefficients, $b\in \mathbb {R}^{pq}$ is an offset term and C is a regularization parameter.

In equation 9, we need to reshape the matrix into vectors which result in losing the correlation among columns or rows in the matrix. By directly transforming the equation 9 for matrix, we get

\begin{equation*} arg \min \frac {1}{2} tr(W^{T}W)+C\sum {1-y_{i}[tr(W^{T}X_{i})+b]}_{+}\tag{10}\end{equation*}

It is known that $tr(WW^{T})=vec(W)vec(W^{T})$ and $tr(W^{T}X_{i})=vec(W)^{T} vec(X_{i})$, thus the above objective function cannot capture the intrinsic structure of each input matrix efficiently, due to the loss of structural information during the reshaping process. To take the advantage of intrinsic structural information within each matrix, one intuitive way is to capture the correlation within each matrix through low-rank constraints on the regression parameter. Recently, Razzak et al. introduced robust support matrix machine (as shown in equation 11) which is a combination of hinge loss and regularization terms ($\ell _{2,1}$ norm and nuclear norm) as spectral elastic net penalty [26]. The regularization terms promotes the structural sparsity and shares similar sparsity patterns across multiple predictors. RSMM is able to maximize the inter-class margins and considers the strong correlation of rows and columns in the matrix, thus, in this work, we have used it for the classification of EEG signls.

\begin{align*}&\hspace {-0.7pc}arg \min \gamma ||W||_{2,1}+\tau ||W||_{*}+C\sum _{}{}{\xi } \\&\qquad w_{j}^{T}x_{i}+b \geq 1-\xi _{i}^{j}, \quad if \: y_{i}=j w_{j}^{T}x_{i}+b \leq -1+\xi _{i}^{j}, \\&\qquad if \: y_{i}\neq j \xi _{i}^{j} \geq 0\tag{11}\end{align*}

where $\xi _{i}^{j}={1-y_{i}[tr(W^{T}X_{i})+b]}_{+}$ is the hinge loss, $W\in \mathbb {R}^{pq}$ is the vector of regression coefficients, $b\in \mathbb {R}^{pq}$ is an offset term and C is a regularization parameter.

## IV. Results and Evaluation

The main goal of this work is to elucidate the best comparable performance as compared to state of the art approaches. In this experiment, we have used four evaluation measures to compare the performance of proposed approach with seven state of the art approaches on four publicly EEG data-sets. In this section, we describe the experimental setup and evaluate the feature selection on EEG classification. As our objective is matrix data classification, thus, for evaluation purpose, we have used datasets where the data is naturally in the form of matrix and structural information is very important such i.e. voltage fluctuations of EEG signal have very strong correlation with respect to certain frequency band and channels. We used different types of publicly available benchmark real-world datasets for EEG classification, we have used two three EEG classification datasets BCI-III IVa, BCI-VI 2a and BCI-VI 2b. The summary of datasets is described Table 2 II-A. Notice that, the dimension of data is much higher than the number of images with in training set for vector classification due to reshaping the matrix data into vectors. This makes the data classification task not only complex but also affect the classification accuracy.

**TABLE 1:** Summary of Dataset

Dataset	subjects	Dimensions	Training Set	Test Set
BCI VI 2a	54	$240\times150$	72	72
BCI III IVa	5	$120\times300$	140	140
BCI VI 2b	9	$150\times24$	200	160

**TABLE 2:** Classification Performance (Accuracy) of Different Algorithms on Dataset BCI 2B

Classifier	SVM	SVM	SVM	SMM	SMM	SMM	SMM	SMM	SMM	RSMM	RSMM	RSMM
Subject/Feature	BCI win	PCA	RPCA	2DPCA	2DRPCA	RPCA	JGSPCA	ORPCA	RJSPCA	RPCA	ORPCA	RJSPCA
S1	0.60	0.68	0.73	0.69	0.69	0.68	0.68	0.71	0.72	0.72	0.73	0.74
S2	0.40	0.50	0.53	0.51	0.51	0.51	0.52	0.52	0.54	0.55	0.56	0.55
S3	0.21	0.52	0.54	0.53	0.51	0.53	0.53	0.51	0.54	0.55	0.56	0.56
S4	0.95	0.91	0.91	0.92	0.87	0.93	0.93	0.95	0.93	0.95	0.97	0.96
S5	0.86	0.8	0.83	0.82	0.80	0.84	0.83	0.84	0. 86	0.87	0.88	0.87
S6	0.61	0.73	0.82	0.76	0.79	0.74	0.75	0.74	0.76	0.78	0.79	0.82
S7	0.56	0.69	0.76	0.75	0.72	0.71	0.72	0.71	0.75	0.76	0.78	0.77
S8	0.85	0.82	0.91	0.87	0.85	0.86	0.83	0.90	0.90	0.90	0.92	0.92
S9	0.74	0.74	0.84	0.77	0.78	0.76	0.76	0.79	0.81	0.84	0.83	0.86
Avg.	0.67	0.71	0.76	0.74	0.72	0.73	0.73	0.75	0.74	0.76	0.78	0.78

To validate the effectiveness of the proposed classifier, we extensively evaluate the proposed approach and compare it with both vector based classifiers (i.e. SVM [4], [8], Sparse SVM (SSVM) [44], LSVM [19], BSVM [12]) as well as with state of the art matrix based classifiers.

### A. Dataset

In this experiment, we have used four publicly available benchmark data-sets namely IIIa,^1^ IVa^2^ of of BCI competition III and IIa,^3^ IIb^4^ of BCI competition IV. IIIa consisted of 60 channel single trial EEG signal obtained from three subjects(k3b, k6b and l1b) while performing four classes of motor imagery (left-hand, right-hand, foot and tongue labeled as class 1, 2, 3 and 4 respectively). IIIa consisted of 45, 30, 30 trials per class for subject k3b, k6b and l1b respectively. Dataset IVa consist of 128 channel, recorded in four session without feedback from five healthy subjects sitting on comfortable chair with arms resting on armrests while performing right-hand or foot motor imagery. IVa consist of 280 trials for each subject with sample rate 100Hz. Similarly, IIa data-set collected in two sessions from nine subjects performing four classes of motor imagery (left-hand, right-hand, foot and tongue). IIa consisted of 288 in total (72 trails per motor imagery). It consisted of 22 EEG channels and 3 monopoloar EOG channels. IIIa and IIa are sampled with 250 Hz and band-pass filtered between 0.5 Hz and 100 Hz. In this experiment, we have considered two subjects (k6b and l1b) for IIIa data-set and EEG channel for IIa dataset. To evaluate, we transformed the multiclass classification problem into binary class problem and generated $C_{4}^{2}=6$ binary subjects namely, L-vs-R, L-vs-F, L-vsT, R-vs-F, R-vs-T and F-vs-T. The dataset IIb is also collected in five sessions (first two without feedback and last three with feedback) from 9 right-handed subjects having normal vision, sitting 1m away from flat screen monitor. The IIb dataset of BCI competation IV was collected from nine subjects while performing left-hand or right-hand motor imagery. IIb consist of 400 trials that are recorded at 3 bipolar channel with sampling rate 250Hz. Thus, in total, we have 71 EEG subject.^1^http://www.bbci.de/competition/iii/#download^2^http://www.bbci.de/competition/iii/#dat_set_iva^3^http://www.bbci.de/competition/iv/#dataset2a^4^http://www.bbci.de/competition/iv/#dataset2b

### B. Evaluation Metrics

In order to evaluate the performance of proposed classifier, we employed different evaluation metrics such as kappa coefficient, precision, recall and F-measure. Furthermore, we have also compared the training time with state of the art approaches. Kappa measure provide evaluation comparison as it consider the the accuracy occurring by chance better. higher the value of k means gain is better classification performance and $k>0$ shows the gain is better than random guess. It is defined as $k=\frac {accuracy-p_{o}}{1-p_{o}}$. Here, $p_{o}$ is the random guess i.e. for a k-class dataset with balanced sample sizes among different classes, we have $p_{o}=\frac {1}{k}$. The other evaluation measures we have used are precision, recall and F-measure. Precision is a measure of classification relevancy i.e. low precision indicates many false positives. Recall is measure of classification completeness and low recall indicates many false negatives. F Score or the F Measure is the weighted harmonic mean of precision and recall.

Furthermore, in this experiment, we have performed k-cross validation (k = 5) to see the generalization of the results by randomizing partitioning the data in to five equal size set and used four set to train classifier and one is used for validation to evaluate the model. The approach is repeated five times such that each of the ten subsets is used exactly once as the validation data.

We first evaluted the performance on BCI-IV-2a EEG dataset of BCI competition-IV. BCI-IV 2a dataset consists of EEG data from 9 healthy subjects recorded in two different sessions performing four classes of motor imagery (left-hand, right-hand, foot and tongue labeled as class 1, 2, 3 and 4 respectively). There are 72 trials per motor imagery task and 288 trials in total per session for each individuals.

### C. Results

Motor imagery-based BCI, which translates the mental imagination of movement to commands, is the huge inter-subject variability with respect to the characteristics of the brain signals [2]. Furthermore, poor characteristics of EEG data such as measurement artifacts, outliers and non-standard noises make it challenging task. In order to reduce the variations, spatial filtering has prevent itself as an effective method for extraction of features has been used as a preprocessing technique to explore the discriminative spatial patterns and eliminate uncorrelated information. In this paper, we have used Filter Bank Common Spatial Pattern (FBCSP) algorithm [2] to filter out the artifacts and unrelated sensor motor rhythms by performing autonomous selection of discriminative subject-specific frequency range for band-pass filtering of the EEG measurements. To select dominant channels for each motor imagery task, we have applied CSP [15] followed by Time domain parameters for feature selection [23] due to its robust performance [37], [42], [43]. We further applied PCA and its variants to select robust features from time domain parameters. As we have selected binary classifier, thus thus, to evaluate, we transformed the multiclass classification problem into binary class problem and generated $C_{4}^{2}=6$ binary subjects namely, L-vs-R, L-vs-F, L-vsT, R-vs-F, R-vs-T and F-vs-T. We have fed the time domain parameters to support matrix machines for classifications and averaged the classification accuracy of nine subjects for each subset. Table 2 and Table 3 shows the comparative evaluation on BCI EEG dataset. Results showed its strong efficiency in the task of EEG signal classification by outperforming state of the art matrix based classification methods. This is due to the fact that EEG signals are strong correlated and sparse. RSSM leverages the structural information as well as dimensionality challenge and promote structural sparsity and model the intrinsic structure. As a result, the regularization term $\ell _{2,1}$ norm along with the nuclear norm and loss not only helps to avoid the inevitable upper bound for the number of selected features but also combines the property of low-rank and sparsity together. Furthermore, the loss function based on $\ell _{2,1}$ and the nuclear norm could help to overcome the outliers as methods based on $\ell _{2}$ [18] and $\ell _{1}$ [42] are sensitive to outliers.

**TABLE 3:** Comparative Evaluation of Classification Performance of Different Algorithms on IIIA Data-Set

Method	Kappa	Precision	Recall	$F_{1}$ Score
PCA-SVM	0.732	0.768	0.799	0.804
2DPCA-SMM	0.784	0.85	0.838	0.844
JSPCA-SVM	0.871	0.91	0.903	0.906
JGSPCA-SMM	0.782	0.847	0.836	0.841
ORPCA-SMM	0.901	0.911	0.901	0.907
RJSPCA-SMM	0.905	0.906	0.897	0.904
ORPCA-RSMM	0.914	0.926	0.915	0.931
RPCA-RSMM	0.916	0.924	0.920	0.924
RJSPCA-RSMM	0.916	0.927	0.918	0.935

We further evaluated the performance of feature selection methods on BCI-IV 2b EEG dataset used for the detection of motor imagery with left and right hand from nine healthy subjects. For each subject, five sessions are recorded, first two sessions (feedback are not considered) are used for training and last three session (recorded with feedback) are used for classification. The evaluation results of all algorithms on the testing set are reported in table 2 and table III. Results showed that RJSPCA provided better classification accuracy as compared to state of the art matrix classification methods that shows that RJSPCA is powerful in selection of robust features. Further mover, we have noticed that RSSM provide considerably better performance as compared to support matrix machines and support vector machines.

We further evaluated the performance of RSSM on BCI III-IVa dataset. The BCI III-IVa dataset consist of 118-channel EEG signals recorded from five subjects (aa, al, av, aw and ay) sampled at 100Hz. The signals are sampled with 250 Hz and band-pass filtered between 0.5 Hz and 100 Hz. For preoprocessing and feature extraction, we performed same techniques that are applied on BCI-IV 2a EEG dataset. The evaluation results of all algorithms on the testing set are reported in figure 2, figure 3, table 2 and table 3. Results showed that matrix based classifier outperform vector based classifiers on all subjects. In comparison to matrix based methods, ORPCA and RJSPCA achieves best performance.

**FIGURE 1.**
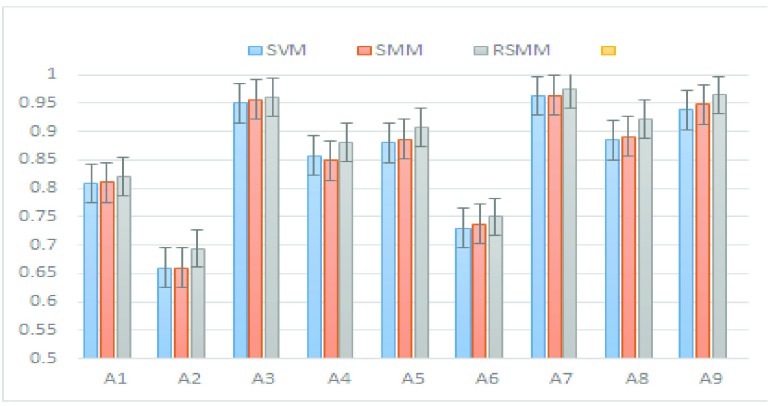
Comparative evaluation of feature extraction methods on IVa dataset.

**FIGURE 2.**
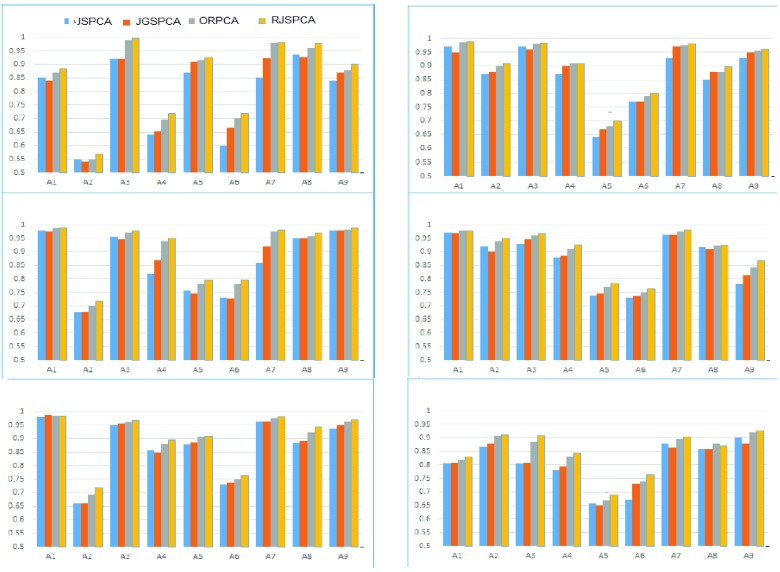
Comparative evaluation of feature extraction methods on IIIa dataset.

**FIGURE 3.**
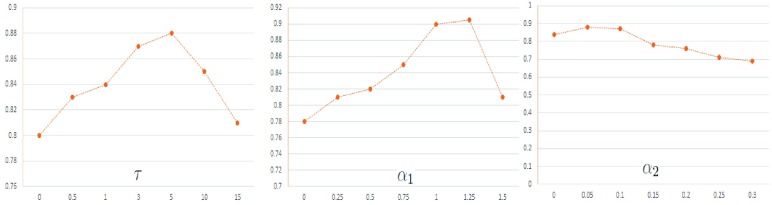
Behaviour of $\lambda $ on on the classification performance for IIa and IIIa datasets.

### D. Discussion

In this section, we provide the comprehensive analysis of feature selection and classification methods. Notice that, the JGSPCA and RJSPCA achieved better performance as compared to the state of the art feature selection methods. he proposed framework not only results in high classification performance but also low FAR. Comprehensive evaluation on benchmark EEG dataset showed that dimensioanlity reduction and classification that directly based on matrix are much better than vector based methods. Figure 3 and Table 2 shows the comparative evaluation on BCI competition IIIa and BCI competition IVa respectively. Notice that, methods based on vector showed poor performance in comprasion to matrix based methods. This is due to the loss of structural information that exisit in the data. Moreover, methods based on vector are computationally complex and feature dimensions are high due to reshaping of matrix into vector. From matrix based methods, feature selection using RJSPCA and ORPCA provided better classification rate. This is due to the robust and joint feature selection for RJSPCA. Similarly, robust PCA that help to deal with outliers through matrix recovery. This shows that RJSPCA and ORPCA suppress the role of outliers by joint feature selection and low rank minimization respectively. The proposed approach reveals the geometric structure due to the fact that it select the features by maintaining the spatial structural information of the image. In terms of results both RJSPCA and ORPCA based classification are comparable, however, RJSPCA is better in term of computational complexity. In results, we can say, that matrix based methods such as ORPCA, RJSPCA are able to finds the representative features from high-dimensional space that are used for classification. It reveals the geometric structure embedded in the data due to the fact that it select the features by maintaining the spatial structural information of the matrix. Figure 3 shows the behaviour of sparse and low rank on classification performance by capturing the correlation of data matrix.

## V. Conclusion

In this work, we validated that direct matrix based classification improves the classification performance as vector based methods ignore the topological structure embedded in the matrix data. We performed evaluation on benchmark EEG datasets. The proposed framework not only results in high classification performance but also low FAR. Comprehensive evaluation showed that dimensioanlity reduction and classification that directly based on matrix are much better than vector based methods. Results showed that RJSPCA and ORPCA provided better classification rate. This is due to the robust and joint feature selection for RJSPCA. Similarly, robust PCA that help to deal with outliers through matrix recovery. In terms of results both RJSPCA and ORPCA based classification are compareable, however, RJSPCA is better in term of computational complexity.
